# Management of Surgical Transcatheter Aortic Valve Replacement (TAVR) Valve Explantation for Early Degeneration: A Case Report

**DOI:** 10.7759/cureus.72143

**Published:** 2024-10-22

**Authors:** Chikashi Nakai, Eduardo Danduch, Saeed Tarabichi, Li Zhang, Sanjay A Samy

**Affiliations:** 1 Cardiothoracic Surgery, Albany Medical Center, Albany, USA

**Keywords:** balloon expandable tavr valve, permanent pacemaker (ppm), surgical aortic valve replacement (savr), tavr (transcatheter aortic valve replacement), tavr valve explantation

## Abstract

Early degeneration of the bioprosthetic aortic valve after transcatheter aortic valve replacement (TAVR) requires aortic valve reintervention, especially in young patients. However, the management of aortic valve reintervention after TAVR is not established. In this case report, the authors report a young patient with an early degenerated TAVR valve who underwent TAVR valve explantation and surgical aortic valve replacement with a mechanical valve. Subsequently, the patient required a permanent pacemaker for a complete heart block. This case report provides one of the management options for early degeneration of the TAVR valve, including benefits and risks from TAVR valve explantation.

## Introduction

Transcatheter aortic valve replacement (TAVR) is increasingly performed in low-surgical-risk and younger patients with a longer life expectancy [1]. All bioprosthetic valves degenerate over time and need to be replaced with valve-in-valve TAVR or surgical aortic valve replacement (SAVR) with TAVR valve explantation. However, the surgical risks of TAVR explantation are higher than isolated first-time SAVR [2]. We experienced a case where the young patient had an early failed TAVR valve and underwent SAVR with a mechanical valve after TAVR valve explantation, subsequently requiring permanent pacemaker (PPM) placement.

## Case presentation

**Figure 1:**
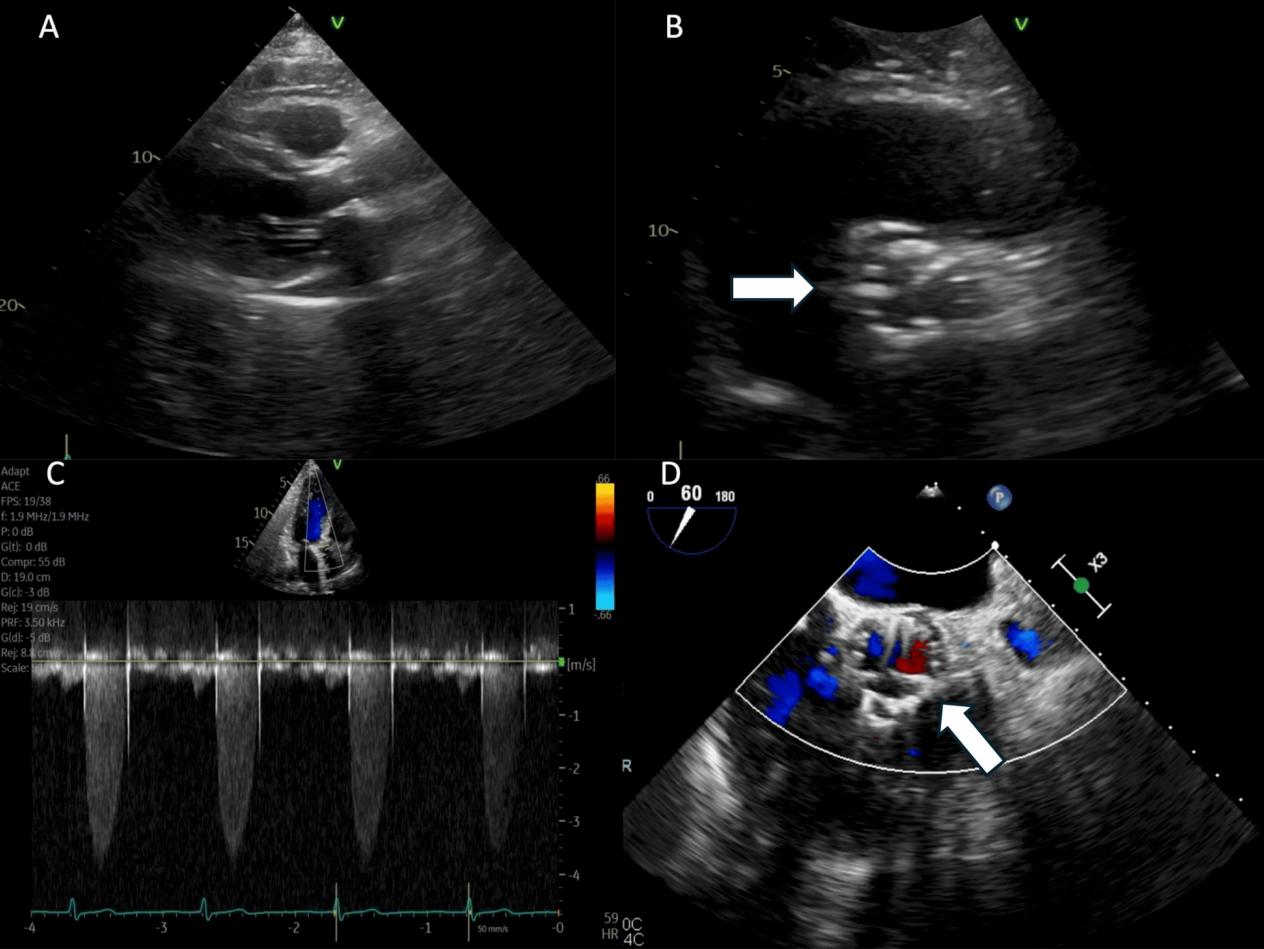
Preoperative transthoracic echocardiogram Normal left ventricular ejection fraction, >55%. 1B, 1C: Restricted leaflet motion (white arrow), mean pressure gradient 34 mmHg, aortic valve area 0.9 cm^2^, 1D: No significant aortic insufficiency (white arrow)

**Figure 2:**
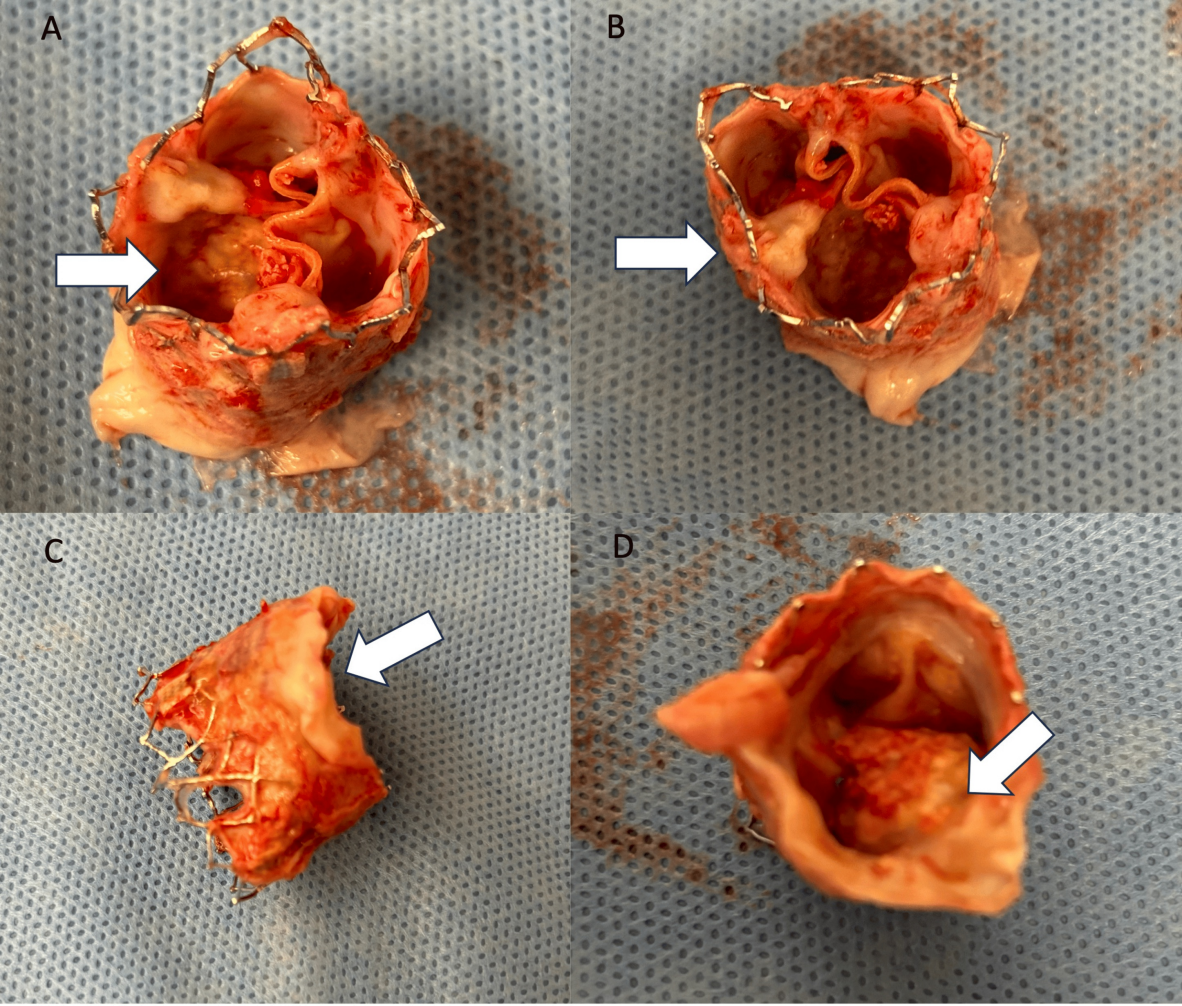
Explanted TAVR valve 2A, 2B: Severely calcified leaflets (white arrow), 2C: Adhesion tissue between the native aortic annulus and the outer skirt of the Sapien 3 valve (white arrow), 2D: View from the left ventricular side, severe calcifications extending from the leaflet to the outer skirt of the Sapien 3 valve (white arrow)

A 65-year-old female presented to the valve clinic with fatigue and dyspnea on exertion for the last three months. Past medical history included severe aortic stenosis (AS), status post-TAVR with a 26 mm Sapien 3 balloon-expandable valve (Edwards Lifesciences, Irvine, CA, USA) four years ago. Transthoracic echocardiogram revealed a preserved ejection fraction (>55%) (Figure 1A) and severe bioprosthetic AS with a mean pressure gradient of 34 mmHg and an aortic valve area of 0.9 cm^2^ (Figures 1B, 1C), without significant aortic insufficiency (Figure 1D). After a discussion about two interventional options for a stenotic TAVR valve including valve-in-valve TAVR and SAVR, she eventually decided to proceed with TAVR valve explantation and SAVR with a mechanical valve to avoid repetitive early degeneration of the TAVR valve. She underwent standard sternotomy, arterial cannulation to the ascending aorta, and venous cannulation to the right atrium to establish a cardiopulmonary bypass (CPB). After the cardiac arrest with antegrade cardioplegia, the aortotomy was performed. The Sapien 3 TAVR valve was located at the aortic annulus position with severely calcified leaflets (Figures 2A, 2B) and severe adhesion between the right coronary sinus and the outer skirt of the valve (Figure 2C). Calcifications extended from the leaflets to the outer skirt of the Sapien 3 valve (Figure 2D). The adhesion and calcification around the aortic annulus were carefully dissected and the valve was subsequently explanted from the annulus. A one cm length of intima tear occurred in the right coronary sinus when the valve was explanted. The tear was repaired with a 5-0 polypropylene stitch. A 23 mm mechanical valve was implanted at the supra-annular position. Once the aortic cross-clamp was removed, she presented with a complete heart block. Epicardial temporary pacing wires were placed on the right atrium and ventricle. The cardiopulmonary bypass (CPB) was weaned and came off with atrioventricular pacing. The CPB and aortic cross-clamp times were 134 and 105 minutes, respectively. The capturing of temporary pacing wires became unstable after the chest closure. The decision was made to proceed with the placement of PPM. PPM was successfully placed in the operation room. She was discharged home on postoperative day 5 without other postoperative complications.

## Discussion

In our case, the patient had degeneration of the TAVR valve with severe calcifications on the leaflets and commissures after four years from the initial TAVR. Calcifications, mechanical degeneration, and immune rejection are the three main mechanisms responsible for structural valve degeneration (SVD) [3]. SVD and pannus ingrowth may cause stenosis or regurgitation, leading to short durability [3]. 

The new-generation TAVR valves have an exterior sealing skirt and may increase tissue incorporation [2]. The structure might cause severe adhesion between the TAVR valve and native aortic annulus tissue [2]. Those technical concerns for dissection in TAVR valve explantation might induce a high incidence of ascending aorta replacement, aortic root replacement, and permanent pacemaker placement [2]. In our case, the dissection between the aortic annulus and TAVR caused a tear of the intima in the right coronary sinus, which was small, and we could avoid aortic root replacement. However, the patient subsequently required PPM for a complete heart block due to the dissection of severe adhesion between the right coronary sinus and the outer skirt of the Sapien 3 TAVR valve. Bapat et al. reported the incidence of new PPM placement after TAVR valve explantation was 18.4% [2]. If the patient underwent SAVR initially, we would not need to do TAVR valve explantation and the intima repair; additionally, we might have been able to avoid PPM.

Landes U et al. reported that the redo TAVR was a safe and effective option for selected patients with failed TAVR valves [4]. If a bioprosthetic valve is implanted in young patients with SAVR or TAVR, many are predicted to require more than one aortic valve intervention [5]. Yerasi C et al. suggested the risks and benefits of three potential strategies in the lifetime management of severe AS in young patients, including SAVR-TAVR-TAVR, TAVR-SAVR-TAVR, and TAVR-TAVR-TAVR [5]. Younger patients must be concerned about the repetitive early degeneration of the TAVR valve even if patients undergo a valve-in-valve TAVR for SVD. In some cases, the valve-in-valve procedure is technically challenging for anatomical problems, including coronary obstruction [2]. In our case, the valve-in-valve TAVR procedure was feasible anatomically. However, the patient decided to proceed with TAVR explantation and replace it with a mechanical valve because of concerns for repetitive early valve failures and potential future aortic valve interventions. TAVR explantation and SAVR with a mechanical valve for SVD may be one of the options in young patients with early SVD and low risk for lifelong anticoagulation. Finding a balance between the risk of bleeding with lifelong anticoagulation with mechanical valves and the risk for reintervention due to SVD of the TAVR valve is important [5]. Younger patients who are expected to require aortic valve reintervention in the future should be carefully evaluated for TAVR candidacy [1]. Young patients who are not good candidates for mechanical valves, have a bicuspid valve, or have a low origin of the coronary ostium may benefit from SAVR first, considering the valve-in-valve TAVR procedure for the future. Candidate selection needs to be crucial for the structural heart team, and the team should consider lifetime management when planning the valve type and approach [1].

## Conclusions

Transcatheter aortic valve replacement for young patients may present with early valve degeneration, and surgical explantation increases postoperative complications. A lifetime strategy for aortic valve intervention needs to be considered when the type of aortic valve is selected.
